# Prediction of 2-year work participation in sickness absentees with neck or shoulder pain: the contribution of demographic, patient-reported, clinical and imaging information

**DOI:** 10.1186/s12891-019-2906-4

**Published:** 2019-11-09

**Authors:** Line Thorndal Moll, Anne Mette Schmidt, Christina Malmose Stapelfeldt, Merete Labriola, Ole Kudsk Jensen, Morten Wasmod Kindt, Tue Secher Jensen, Berit Schiøttz-Christensen

**Affiliations:** 1 DEFACTUM, P.P. Oerums Gade 11, bygn. 1B, Aarhus C, DK-8000 Aarhus, Central Denmark Region Denmark; 2 Section of Clinical Social Medicine and Rehabilitation, Department of Public Health, Aarhus University, P.P. Oerums Gade 9-11, bygn. 1B, Aarhus C, DK-8000 Aarhus, Denmark; 3 Spine Centre, Diagnostic Centre, Silkeborg Regional Hospital, Falkevej 1–3, DK-8600 Silkeborg, Denmark; 4 Sano Aarhus, Egernvej 5, DK-8270 Højbjerg, Aarhus, Denmark; 5 Department for Diagnostic Imaging, Diagnostic Centre, Silkeborg Regional Hospital, University Research Clinic for Innovative Patient Pathways, Falkevej 1-3, DK-8600 Silkeborg, Denmark; 6 Nordic Institute of Chiropractic and Clinical Biomechanics, University of Southern Denmark, Campusvej 55 Odense M, DK-5230 Odense, Denmark; 7 Spine Centre of Southern Denmark, Hospital Lillebaelt Middelfart and Institute of Regional Health Research, University of Southern Denmark, Oestre Hougvej 55 Middelfart, DK-5500 Odense, Denmark

**Keywords:** Prognosis, Prediction, Sick leave, Neck pain, Shoulder pain, Magnetic resonance imaging

## Abstract

**Background:**

In cases of neck and shoulder pain, the responsibility for assessing work prognosis is held by clinicians with access to different domains of information. One of these domains is magnetic resonance imaging (MRI), and although MRI is increasingly used, it is unknown which domains of information contribute the most to the prediction of work prognosis. This retrospective cohort study explored the contribution of demographic, patient-reported, clinical, and MRI information to the prediction of work participation in sickness absentees with neck or shoulder pain.

**Methods:**

From a secondary care setting, 168 sickness absentees with neck or shoulder pain were included. Based on registry data, a successful work outcome was defined as ≥50% work participation score (WPS) from Weeks 1 to 104 after enrolment. Prognostic variables were categorized into four domains (demographic, patient-reported, clinical, and MRI) resembling the order of information obtained in a clinical setting. Crude logistic regression analyses were used to identify prognostic variables for each domain (*p* < 0.2). This was followed by multivariable analyses including the identified variables in a domain-wise order. For each added domain, the probability of successful WPS was dichotomized leaving two possible classifications: ≥ 50% chance of successful WPS or not. In cross-tabulations of chance and the actual WPS outcome, positive and negative predictive values (PPV and NPV), sensitivity, specificity and area under the curve (AUC) were calculated.

**Results:**

The combination of demographic and patient-reported variables yielded an NPV of 0.72 and a PPV of 0.67, while specificity was 0.82, sensitivity 0.54 and AUC 0.77. None of these values improved notably by adding clinical and MRI variables as predictors of successful WPS.

**Conclusions:**

These results suggest that - among sickness absentees with neck or shoulder pain – clinical and MRI variables provide no additional information for the prediction of work participation compared with only demographic and patient-reported information.

## Background

During sick leave, estimating work prognosis is important as it helps guide expectations for all involved stakeholders. The responsibility for this usually resides with healthcare professionals and it is often requested of them by authorities involved in return-to-work (RTW) coordination and the disbursement of social benefits, i.e. sickness benefits. When assessing work prognosis, healthcare professionals have access to different domains of information. In a clinical setting, such information is typically obtained in the following order: 1) demographics are known already when scheduling the clinical encounter, and are followed by 2) patient-reported information which is a necessary prerequisite for performing 3) a focused clinical examination, and deciding whether 4) imaging is needed (e.g. magnetic resonance imaging (MRI)).

From this range of information, the literature offers insight into known prognostic factors. From the demographic domain, older age is negatively associated with RTW [1, 2], while for the impact of sex, results are conflicting [1–3]. Patient-reported information that is negatively associated with work outcomes includes symptom intensity, sick leave duration [1, 3], ongoing workers’ compensation claim [4] and high physical work demands [1]. From the clinical domain, low back pain studies have found a negative association between radiating pain and RTW in the acute phase (< 6 weeks) [5] and moderate evidence for no association with RTW in the sub-acute and chronic phases [2]. Also from the clinical domain, fibromyalgia is associated with poor work participation [6]. From the MRI domain, a single study involving sickness absentees with low back pain found Modic type 1 changes on MRI to predict unsuccessful RTW [7].

However, the prognostic value of imaging has been described as “a gap in the literature” [8]. This gap still deserves investigation as the number of MRIs has increased more than the prevalence of people suffering from neck and shoulder pain [9–11]. Moreover, the referral patterns have changed over recent years; e.g. in Denmark, not only hospital physicians but also primary care clinicians (physicians and chiropractors) can request MRI. For clinicians in both primary and secondary care, this change in MRI referral patterns has changed the range of information available when estimating work prognosis.

The literature thus offers insight into factors associated with work prognosis. But to our knowledge, no previous studies have examined to what extent the different domains of information contribute to the prediction of work prognosis. Thus healthcare professionals’ assessment of work prognosis may be based on gut-feeling rather than evidence. Therefore, the aim of this study was to explore the degree to which demographic, patient-reported, clinical, and MRI information contribute to the prediction of work participation in sickness absentees with neck or shoulder pain.

## Methods

### Design and participants

Using a retrospective cohort design, 168 sickness absentees referred to secondary care for assessment of neck or shoulder pain were included in this study. Neck pain was defined as pain perceived anywhere in the posterior region of the cervical spine. Neck pain may refer to the head, the shoulder, the anterior chest wall or the upper limb [12]. Shoulder pain was defined as pain perceived around the shoulder; it may originate from the neck, any of the three joints adjacent to the shoulder (glenohumeral, acromioclavicular and sternoclavicular joints) or adjacent soft tissues [13]. All participants were part of an RCT [14] that took place from 2009 to 2014 and compared the work outcomes after two different hospital-based interventions (multidisciplinary vs. brief intervention). The inclusion criteria for the RCT were: sick leave 1–4 months owing to neck or shoulder pain, age 18–60 years, and fluency in Danish. Exclusion criteria were: alcohol or drug addiction, pregnancy, specific musculoskeletal/neurological disease, surgery within the past year, plans for surgery, and primary psychiatric disorder. Further details regarding recruitment and interventions have been described previously [14].

### Context (Danish legislation on sickness benefits)

According to The Danish Sickness Benefit Act, sickness absentees are entitled to financial compensation during sick leave. This compensation can amount to sickness benefits or full wages depending on the union award conditions the worker is entitled to. At the time of the RCT (2009–2014), the employer covered these expenses for the first 21–30 days of a sick leave spell (called the employer paid period) after which the municipality reimbursed some of the employer’s expenses (the sickness benefit amount) until RTW was achieved. In Denmark, a special agreement (§56) can be settled upon in cases of recurrent short-term sick leave; it entitles the employer to be reimbursed from the first day of sick leave, thereby reducing the employer’s expenses [15].

### Outcome: work participation score (WPS)

Data on work outcomes were supplied by the Danish Register for Evaluation of Marginalization (DREAM) which covers the source of income (financial self-support or public transfer benefits) on a weekly basis since July 1991. The registrations are ordered hierarchically; if different transfer benefit codes are registered in the same week, the highest-ranking code will overwrite the others. Sickness benefits have a high rank and are only overwritten in cases of emigration, age-related pensions or death [16].

The work participation score (WPS) is a fraction yielding scores between 0 and 100% [17]. The numerator comprises the number of weeks with financial self-support (interpreted as working weeks) as well as weeks with state education fund grants and related benefits (benefits granted in cases of re-education). The denominator comprises the total number of follow-up weeks [17]. In this study, three individuals were granted early retirement. For these three, the denominator comprised the number of follow-up weeks prior to the first appearance of retirement benefits in DREAM. Follow up started at the day of inclusion in the original RCT [14], and a successful outcome was defined as a WPS ≥ 50% during weeks 1–104 after enrollment. The 50% threshold was chosen because it has shown substantial to almost perfect agreement compared with other RTW measures [17]. The following denotations were used: successful WPS (s-WPS) ≥ 50% and unsuccessful WPS (u-WPS) < 50%.

### Prognostic variables: demographic and patient-reported domain

These data were collected from questionnaires filled in by the participants prior to their first meeting with the health care professionals in the RCT [14]. The information provided covered demographics (sex and age) and the following patient-reported measures: pain intensity (11-point numeric rating scale) [18], whiplash trauma (yes/no) [19], education (≥ 3 years, yes/no), worker’s compensation claim (yes/no) [20], and Örebro Musculoskeletal Pain Screening Questionnaire (ÖMPSQ) [21]. The ÖMPSQ score covers psychosocial risk factors of work disability (e.g. coping abilities, pain beliefs, disability, feelings of anxiety or depression and self-perceived chances of returning to work in 6 months. The ÖMPSQ was originally developed as a screening tool to identify psychosocial risk factors for prolonged disability and sick leave; the higher the score (maximum = 210), the higher the risk of prolonged disability.

### Prognostic variables: clinical domain

Data on radiculopathy and number of tender points [22, 23] came from the clinical examination performed by the rheumatologist prior to enrolment in the RCT [14]. Radiculopathy describes a clinical assessment based on 1) pain radiating from the neck to the upper extremity and 2) one or more positive neurological signs: weakened deep tendon reflexes, decreased muscle strength, dermatomal sensory deficits or a positive foraminal compression test. The reliability of the rheumatologist’s tender points count has previously been reported as excellent [24] and a threshold of ≥11 tender points was chosen, since this is still commonly used among clinicians to assess fibromyalgia [25]. In the clinical examination, the tender points count was used as a measure of sensitization of the nociceptive system [26]. Since data were not sufficient to support the diagnosis of fibromyalgia, this term was not used and a dichotomized threshold of the tender points count was used instead. The intervention allocation in the original RCT [14] (multidisciplinary and brief intervention) were also included.

### Prognostic variables: MRI domain

MRI of the cervical spine was ordered for all participants unless clinical examination revealed unambiguous signs of shoulder pathology (*n* = 7). For different reasons, MRI of the cervical spine was only available for 97 of the participants (no access to MRI: *n* = 32 and unsatisfactory signal-to-noise ratio assessed by a senior radiologist: *n* = 32 [27]). All MRIs were de-identified and assessed by the first author (physician with 9 years of post-graduate clinical experience) who was blinded to the study outcomes. The inter-rater reliability of the MRI assessments has previously been reported with un-weighted kappa values ranging from 0.56 to 0.79 [27] while intra-rater reliability yielded kappa values ranging from 0.67 to 0.90 [27]. The MRI variables listed in Table 1 were evaluated at disc levels C2/C3 to C7/T1. For each participant, an MRI finding was defined as positive if the finding was present at ≥1 disc level, i.e. two levels with disc height reduction were counted only once.

**Table 1:** Baseline characteristics for sickness absentees with neck or shoulder pain

	Entire study sample		MRI sample	
Demographics	N		N_MRI_	
Male sex, n (%)	168	53 (31.5)	97	30 (30.9)
Age, median (IQR)	168	40.5 (34.5; 48.9)	97	41.4 (35.3; 49.9)
Patient-reported information				
Pain intensity (0–10) last week, median (IQR)	158	7 (5; 8)	91	7 (5; 8)
Whiplash trauma, n (%)	168	24 (14.3)	97	13 (13.4)
Sick leave duration (months), median (IQR)	168	2.3 (1.2; 3.0)	97	2.3 (1.4; 3.0)
ÖMPSQ score, mean (SD)	161	122 (23.7)	93	121.8 (22.1)
Education ≥3 years, n (%)	155	29 (18.7)	88	21 (23.9)
Ongoing worker’s compensation claim, n (%)	139	36 (25.9)	82	19 (23.2)
Clinical information				
Radiculopathy, n (%)	168	41 (24.4)	97	28 (28.9)
≥ 11 tender points, n (%)	168	57 (33.9)	97	38 (39.2)
Intervention group				
Brief intervention, n (%) Multidisciplinary intervention, n (%)	168	83 (49.4) 85 (50.6)	97	49 (50.5) 48 (49.5)
MRI information				
Kyphosis, n (%)			97	23 (23.7)
Disc height reduction, n (%)			97	71 (73.2)
Bulge, protrusion or extrusion, n (%)			83	69 (83.1)
Spinal canal stenosis, n (%)			97	14 (14.4)
Neural foraminal stenosis, n (%)			84	46 (54.8)
Zygapophyseal osteoarthritis, n (%)			83	39 (47.0)
Uncovertebral osteoarthritis, n (%)			78	37 (47.4)

### Statistical analyses

Crude logistic regression analyses were performed for all baseline variables hypothesized to affect WPS, and variables yielding *p*-values < 0.2 were carried forward as has been done in other studies [28, 29]. In the multivariable analyses, the prognostic variables were included using a domain-wise approach resembling the typical order of information obtained in a clinical setting. First, demographic variables were included, second patient-reported, third clinical, and finally MRI variables – these are referred to as Models 1, 2, 3 and 4. For each model, the probability of s-WPS was calculated and dichotomized, i.e. the participants were classified as having ≥50% chance of s-WPS or not. By comparing the estimated chance of s-WPS with whether s-WPS actually happened or not, sensitivity, specificity, positive and negative predictive values (PPV and NPV), and area under the curve (AUC) were calculated. In this study, sensitivity denotes the proportion of u-WPS correctly classified as *‘< 50% chance of s-WPS’,* and specificity, the proportion of s-WPS that is correctly classified as *‘≥50% chance of s-WPS’*. In accordance with this, NPV denotes the proportion of sickness absentees classified as *‘≥50% s-WPS chance’* who actually achieved s-WPS. PPV denotes the proportion of sickness absentees classified as ‘*< 50% s-WPS chance”* who did not achieve s-WPS. AUC is a measure of discriminatory ability which describes the probability that a worker achieving s-WPS will be classified as having a better chance than a worker not achieving s-WPS. To ensure comparability between the models, estimates were presented both for the number of individuals with complete data and for the number of individuals with complete data in the adjacent model (e.g. in Model 3: estimates were presented for the 139 individuals with complete data on demographic, patient-reported and clinical domains and for the 82 individuals with complete data in Model 4). For each added domain of information, sensitivity, specificity, PPV and NPV were compared between the models. To further appraise the properties of the models, the chance of s-WPS was divided into four categories (< 30%, ≥ 30% and < 50%, ≥ 50% and < 70%, ≥ 70%) and tabulations were made to assess whether adding domains of information improved prediction for those with the lowest (< 30%) and highest (≥ 70%) chance of s-WPS. Estimates were reported using 95% confidence intervals (CIs).

The statistical analyses were performed by the first author who was not involved in the clinical assessment or interventions given in the original RCT [14]. STATA15 statistical software package was used for all statistical analyses (STATA Corp., College Station, Texas, USA).

### Nomenclature

This study explored the associations between prognostic variables and s-WPS. The term “prognostic variable” denotes a baseline variable which is associated with the outcome s-WPS but without making inferences about causality [30]. The terms *‘predict’/'prediction’* relate to the ability of the models to forecast the prognosis regarding work participation.

### Ethics

All participants provided written informed consent. The study was approved by The Danish Data Protection Agency (J. no. 2012–58-006) and by The Central Denmark Region Committees onHealth Research Ethics (M-20090027).

## Results

One hundred-and-sixty-eight sickness absentees were included. S-WPS was achieved by 100 (59.5%) of the sickness absentees, i.e. 59.5% of the sickness absentees had a work participation score ≥ 50% from Week 1 to 104 after enrolment. The proportion achieving s-WPS was similar (59.8%) in the MRI sample. Baseline characteristics are presented in Table 1.

The crude associations between potential prognostic variables and s-WPS are presented in Table 2. For those variables yielding *p* < 0.2, the following associations were seen: Increased odds of s-WPS were found for male sex, radiculopathy and kyphosis. Decreased odds of s-WPS were found for increased sick leave duration (months), increased ÖMPSQ score, ongoing workers’ compensation claim, ≥ 11 tender points, and spinal canal stenosis. The remaining variables from Table 2 did not fulfill the criterion of *p* < 0.2 for inclusion in the multivariable analyses

**Table 2:** Odds ratios for s-WPS. Results from univariable analyses

Demographic information	N	Odds ratio (95% CI)	*p*-value
Male sex	168	1.69 (0.85; 3.34)^a^	0.13
Age (years)	168	0.99 (0.97; 1.03)	0.85
Patient-reported information			
Pain intensity (numeric rating scale)	161	0.98 (0.95, 1.01)	0.21
Whiplash trauma (yes vs. no)	168	0.77 (0.32; 1.85)	0.56
Sick leave duration (months)	168	0.68 (0.54; 0.87)^a^	0.002
ÖMPSQ score (numerical variable)	161	0.97 (0.95; 0.98)^a^	< 0.001
Education ≥3 years (yes vs. no)	155	0.93 (0.41; 2.12)	0.87
Ongoing worker’s compensation claim (yes vs. no)	139	0.45 (0.21; 0.97)^a^	0.04
Clinical information			
Radiculopathy (yes vs. no)	168	4.29 (1.77; 10.4)^a^	0.001
≥ 11 tender points (yes vs. no)	168	0.58 (0.31; 1.12)^a^	0.10
Multidisciplinary intervention	168	1.04 (0.56; 1.93)	0.90
MRI information			
Kyphosis (yes vs. no)	97	3.06 (1.03; 9.11)^a^	0.05
Disc height reduction (yes vs. no)	97	1.40 (0.56; 3.46)	0.47
Disc contour change (bulge, protrusion or extrusion) (yes vs. no)	83	1.32 (0.41; 4.24)	0.64
Spinal canal stenosis (yes vs. no)	97	0.31 (0.09; 1.02)^a^	0.06
Neural foraminal stenosis (yes vs. no)	84	1.13 (0.47; 2.71)	0.78
Zygapophyseal osteoarthritis (yes vs. no)	83	0.81 (0.34; 1.96)	0.65
Uncovertebral osteoarthritis (yes vs. no)	78	1.44 (0.58; 3.60)	0.43

*s-WPS* successful work participation score, ^a^: Variables carried forward in the multivariable analyses, *ÖMPSQ* Örebro Musculoskeletal Pain Screening Questionnaire, *MRI* Magnetic Resonance Imaging

Table 3 presents the predictive values from Models 1–4. As shown, knowledge about only the sex offered an NPV of 0.60 (0.52; 0.67) and a sensitivity of 1.0 (0.96; 1.0) (the 100 sickness absentees who achieved s-WPS were all classified as having ≥50% chance). The PPV could not be calculated since none were classified as having < 50% chance. By adding patient-reported variables (duration of sick leave, ÖMPSQ score and ongoing workers’ compensation claim), the NPV increased to 0.72 (0.62; 0.81) and the PPV to 0.67 (0.52; 0.80) along with increased sensitivity. Adding clinical and MRI information (Models 3 and 4) elicited only minor changes which – based on confidence intervals – did not differ from Model 2.

**Table 3:** Predictive values, sensitivity, specificity and AUC for prediction of work participation in each of the multivariable models

Information included	N	Classified as ≥50% chance *and* achieved s-WPS (NPV)	Classified as < 50% chance *and* achieved u-WPS (PPV)	s-WPS correctly classified (specificity)	u-WPS correctly classified (sensitivity)	*AUC*
Model 1: Demographic^a^	168	0.60 (0.52; 0.67)	NA	1.0 (0.96; 1.0)	0.0 (0.0; 0.05)	0.56 (0.48; 0.63)
Model 1: Demographic	139	0.59 (0.50; 0.67)	NA	1.0 (0.96; 1.0)	0.0 (0.0; 0.06)	0.56 (0.47; 0.64)
Model 2: Demographic + patient-reported^b^	139^‡^	0.72 (0.62; 0.81)	0.67 (0.52; 0.80)	0.82 (0.72; 0.89)	0.54 (0.41; 0.68)	0.77 (0.69; 0.84)
Model 3: Demographic + patient-reported + clinical^c^	139^‡^	0.74 (0.63; 0.82)	0.69 (0.54; 0.81)	0.82 (0.71; 0.89)	0.58 (0.44; 0.71)	0.78 (0.70; 0.89)
Model 3: Demographic + patient-reported + clinical^c^	82	0.71 (0.59; 0.83)	0.72 (0.50; 0.89)	0.87 (0.75; 0.95)	0.48 (0.31; 0.66)	0.72 (0.65; 0.86)
Model 4: Demographic + patient-reported + clinical + MRI^d^	82^‡^	0.74 (0.60; 0.84)	0.72 (0.50; 0.88)	0.86 (0.73; 0.94)	0.55 (0.36; 0.72)	0.80 (0.70; 0.90)

^a^: male sex OR = 1.69 (95% CI: 0.85; 3.34), ^b^: male sex OR = 1.32 (95% CI: 0.58; 3.02), sick leave (months) OR = 0.66 (95% CI: 0.49; 0.88), ÖMPSQ score OR = 0.97 (95% CI: 0.95; 0.99), ongoing workers’ compensation claim OR = 0.46 (95% CI: 0.19; 1.08, ^c^: male sex OR = 1.03 (95% CI: 0.42; 2.57), sick leave (months) OR = 0.66 (95% CI: 0.49; 0.89), ÖMPSQ score OR = 0.97 (95% CI: 0.95; 0.99), ongoing workers’ compensation claim OR = 0.51 (95% CI: 0.21; 1.21), radiculopathy OR = 2.83 (95% CI = 0.99; 8.1), ≥ 11 tender points OR = 0.96 (95% CI: 0.41; 2.22), ^d^: male sex OR = 1.67 (95% CI: 0.48; 5.9), sick leave (months) OR = 0.59 (95% CI: 0.37; 0.94), ÖMPSQ score OR = 0.98 (95% CI: 0.95; 1.0), ongoing wokers’ compensation claim OR = 0.53 (95% CI (0.16; 1.80), radiculopathy OR = 2.27 (95% CI = 0.48; 10.7), ≥ 11 tender points OR = 0.95 (95% CI: 0.31; 2.9), kyphosis OR = 2.78 (95% CI: 0.53; 14.5), spinal canal stenosis OR = 0.09 (95% CI: 0.01; 0.72), *NPV* negative predictive value, *PPV* positive predictive value, *s-WPS* successful work participation score, *u-WPS* unsuccessful work participation score, *AUC* area under the curve, ^‡^: comparison with previous step is supported by similar N on both steps

Additional detail regarding Models 1–4 is offered by Table 4 where the chance of s-WPS in four categories is compared with actual WPS outcomes. Model 1 did not identify anybody with < 30% or ≥ 70% of s-WPS, whereas Model 2 performed better than Model 1, discriminating among those whose chance of s-WPS was very low or very high. The discriminating properties of the models were similar for Models 2, 3 and 4 (assessed by the distribution among categories of chance).

**Table 4:** Cross-tabulations showing the classification of chance vs. the achieved work participation score during weeks 1–104 of follow up

Domains included	N		Outcome	
Demographics	168	Chance of s-WPS	s-WPS	u-WPS
		Chance < 30%	0	0
		30% ≤ chance < 50%	0	0
		50% ≤ chance < 70%	100	68
		≥ 70%	0	0
Demographic + patient-reported	139		Outcome	
		Chance of s-WPS	s-WPS	u-WPS
		Chance < 30%	7	9
		30% ≤ chance < 50%	8	22
		50% ≤ chance < 70%	25	19
		≥ 70%	42	7
Demographic + patient-reported + clinical	139		Outcome	
		Chance of s-WPS	s-WPS	u-WPS
		Chance < 30%	5	14
		30% ≤ chance < 50%	10	19
		50% ≤ chance < 70%	23	17
		≥ 70%	44	7
Demographic + patient-reported + clinical + MRI	82		Outcome	
		Chance of s-WPS	s-WPS	u-WPS
		Chance < 30%	3	7
		30% ≤ chance < 50%	4	11
		50% ≤ chance < 70%	15	12
		≥ 70%	27	3

*WPS* work participation score, s*-WPS* successful work participation score, i.e. WPS ≥ 50% in weeks 1–104, *u-WPS* unsuccessful work participation score, i.e. WPS < 50% in weeks 1–104

## Discussion

To our knowledge, this is the first study exploring the contribution of demographics, patient-reported, clinical and MRI information to the prediction of work participation in sickness absentees with neck or shoulder pain. The results suggest that demographics and patient-reported measures include important prognostic information (sex, duration of sick leave prior to enrollment and ÖMPSQ score) and that neither clinical nor MRI information provide substantial additional information to the prediction of work participation within 2 years. That is, Model 2 performed similarly to Models 3 and 4 regarding sensitivity, specificity, NPV, PPV and AUC. Model 2 also performed similarly to Models 3 and 4 at discriminating between those having the lowest and highest chance of s-WPS (Table 4).

### Comparison with other studies

59.5% of the sickness absentees achieved s-WPS within two-year follow up, which is indicative of a population with a high level of work disability. In a large study of neck and back pain [31], only 8% reported sick leave the previous year when asked at 3-year follow up. The study [31] was conducted in a workplace setting, hence, study participants were presumably less disabled than those referred to secondary care settings as in the current study. From secondary care settings however, previous studies [7, 32] have also found what seemed to be better work outcomes than in the current study. In a study of low back pain, 72% achieved RTW within 1 year; however RTW was defined as 4 consecutive weeks of work [7]. In another study of primarily musculoskeletal pain, 60 % achieved had a more sustainable RTW outcome at 1 year follow up, namely 3 consecutive months with increased working time compared to baseline [32]. The study population of this study [32] was comprised of sickness absentees referred to a secondary care setting like ours. Hence, we consider the similarity of work outcomes to be indicative of work disability that hampers work participation.

A comprehensive literature search strategy adapted from a 2017 review [2] was performed using Medline and yet, no studies were identified which categorized prognostic variables and used an analytical approach resembling the order of information obtained in a clinical setting. For this reason, direct comparison with previous studies was not possible, but some studies reported measures that can be compared with the current study [33, 34]. In a Norwegian study [33], the sickness absentees’ own prediction of sick leave duration ≥26 weeks yielded a slightly better PPV (0.78) than any of the models in the current study. However, the sensitivity based on self-prediction was 0.28 and the sensitivity based on dedicated medical consultants was 0.07. Both measures are notably lower than the sensitivity estimates of the current study suggesting that 1) perhaps the sickness absentees in Fleten et al.’s study were over-optimistic, leading to a low sensitivity of self-prediction, and 2) the information that was available to the medical consultants [33] was inadequate to result in a sensitivity as high as in the current study.

Rehabilitation professionals in another study [34] predicted the chance of RTW based on sick leave duration, reason for sick leave, unemployment, age > 45 years, female sex and ‘gut feeling’. Their prediction was concordant with actual RTW in 73% and thus lower than the specificity estimates of all four models in the current study. Of interest is that the rehabilitation professionals in that study [34] had access to some similar information as was available in the current study, namely sex and sick leave duration. But in spite of their access to further information about reason for sick leave, unemployment, age > 45 and “gut feeling”, specificity estimates similar to the current study were not achieved.

Regarding the association between sex and work outcomes, results in previous studies have been conflicting. In a population-based study [35] and in studies from secondary care on spinal pain [36] and shoulder pain [37], sex was not associated with work outcomes, while a primary care study [28] did find predictive value of sex. In summary, reviews have found conflicting evidence on the impact of sex on work outcomes [1, 3, 28, 35–37] and with this in mind, the current study’s estimates in Model 1 based on sex alone are not surprising.

The impact of sick leave duration corroborates the findings in numerous reviews [1, 3, 8, 38, 39] and cohort studies of both shoulder pain, spinal pain and whiplash trauma [28, 40, 41]. Due to the non-modifiable nature of sick leave duration, our findings highlight the need for action that prevents and/or addresses sick leave early enough to minimize the duration.

The ÖMPSQ score covers psychosocial risk factors of work disability (e.g. feelings of anxiety or depression and self-perceived chances of returning to work in 6 months). The negative association between the ÖMPSQ score and s-WPS is in line with previous findings [21, 42]. While studies from primary care settings and workplaces [43, 44] have suggested ÖMPSQ cut-off values of 90 and 105, the current study suggests that in a secondary care setting, ÖMPSQ has important properties regarding prognosis of work participation also as a discrete variable.

Interestingly, a study of primarily chronic spinal pain suggested cut-offs of 90 and 105 [44] but did not yield sensitivity and specificity measures that were concurrently as high as any of the models including ÖMPSQ in the current study. In contrast, a primary care study of patients with acute or sub-acute spinal pain [43], a cut-off of 90 yielded better sensitivity and slightly lower specificity [43], thereby demonstrating the properties of ÖMPSQ in early detection of poor work prognosis.

The negative association between ongoing workers’ compensation claim and s-WPS is in line with the findings of a previous Danish study of patients with neck/arm pain or back/leg pain [4].

In the context of clinical variables, a cohort study on low back pain [45] found a crude association between tender points count (discrete variable) and unsuccessful RTW. However, this was not maintained in the multivariable model. Moreover, no association was found between radiculopathy and work outcomes [45], which was supported by a review presenting moderate evidence for no association between radiating pain and RTW [2]. Therefore, our finding of the limited improvement of predictive values in Model 3 is in line with these previous findings [2, 45].

Finally, regarding MRI, only one study was identified which explored the association between MRI findings and work outcomes [7]. In this study of sickness absentees with low back pain, the presence of Modic type 1 changes was associated with unsuccessful RTW. Unfortunately, we were unable to assess the impact of Modic changes in the current study since the inter- and intra-rater reliability for this pathology was not established owing to too low prevalences [27].

### Methodological considerations

WPS was chosen as the outcome measure in an attempt to capture both work reintegration and work maintenance although we are aware that other RTW outcomes reflect other aspects regarding the process and context of RTW [46]. The chosen cut-off value (s-WPS ≥ 50% or not) showed substantial to almost perfect agreement with other RTW measures (RTW yes/no at a given time point and time to 4 weeks of self-support without relapses, respectively) in a previous study [17]. The same study [17] found WPS suitable for prognostic purposes. When classifying the chance of s-WPS, a 50% threshold was chosen for two reasons: 1) A reasonable balance between sensitivity and specificity measures was desired and 2) Our clinical experience suggests that sickness absentees with neck or shoulder pain who are seen in secondary care are troubled with many biopsychosocial issues. Hence, choosing higher cut-offs for the chance of s-WPS might be too optimistic. A different cut-off would probably be relevant if the study population had comprised patients from primary care on short-term sick leave.

Inclusion of more variables (e.g. more patient-reported, clinical or occupational information) was not possible owing to power considerations.

### Strengths

First, the use of registry data for the outcome assessment ensured 100% follow up thereby reducing the risk of attrition bias. Second, the use of registry data reduced the risk of measurement bias since the outcome assessment was unaffected by knowledge of the prognostic variables. Likewise, the assessment of prognostic factors was unaffected by knowledge of the outcome. Third, the risk of attrition bias was minimized by the low number of missing values for the majority of variables (demographic, patient-reported and clinical variables). Only for MRI was the number of missing values substantial. However, the distribution between exposure and outcome was assessed for all the variables in Table 1, which revealed that attrition was not skewed for any of the variables, hence attrition bias was not suspected (data not shown). Fourth, the duration of follow up reaching 2 years constitutes a realistic long-term outcome. A fifth strength is the a priori decision to take an analytical approach resembling the working conditions of the clinicians who are responsible for appraisal of work prognosis. This reduced the risk of purely data-driven results. Sixth, the risk of bias owing to potential misclassification of MRI findings was minimal owing to high levels of observed agreement for kyphosis and spinal canal stenosis [27]. Finally, when bearing in mind the well-established impact of external societal factors on the process of sick leave [47, 48], it is a further strength of the current study that the Danish legislation on sickness benefits did not undergo major changes from 2009 to 2014. A change in legislation in 2014 reduced the right for sickness benefits from 52 to 22 weeks. It affected only six sickness absentees (four achieved s-WPS and two did not) and is thus not suspected to bias the estimates.

### Limitations

First, since the study population was originally included in an RCT, the results may not be generalizable to all sickness absentees with neck or shoulder pain. The possibility of sampling bias due to referral patterns of the general practitioners cannot be refuted nor elucidated. Hence, the results are expected to be representative of sickness absentees with neck or shoulder pain who are seen in a secondary care setting. Generalizability to primary care settings should be made with caution and confirmatory studies including all sickness absentees with neck or shoulder pain would be needed to improve generalizability.

Second, the representativeness of the results should be considered in the light of the above-mentioned change in legislation, implying that the right to sickness benefits is now limited to 22 weeks compared with 52 weeks during most of the study. Confirmatory studies would be desirable to assess the impact of this change in legislation. But given that the literature over the past decades is corroborated (i.e. major impact of sick leave duration and psychosocial factors [49]), we expect that similar results would be found.

Third, the limited MRI sample affects precision of the estimates; an issue that was further attenuated for certain MRI variables (disc bulge/protrusion/extrusion, neural foraminal stenosis, zygapophyseal osteoarthritis and uncovertebral osteoarthritis). For these variables, the available sequences did not allow for evaluation on all 97 MRIs (Table 1 and [27]). Had MRI been available for the entire cohort, confidence intervals for MRI variables would have been narrowed down.

Fourth, the use of registry data is usually an advantage, but the data source also warrants consideration since registration procedures imply a risk of unequal registration of short-term sick leave. Sick leave registration in DREAM begins at the end of the employer paid period and backward adjustments are made, so that the number of sickness benefit weeks in DREAM equal the total number of sick leave weeks. Since registration is initiated at the end of the employer paid period, multiple absences due to sick leave lasting only days or a few weeks are usually not registered (because they are within the employer-paid period). In the current study, some participants may have been misclassified as having achieved s-WPS although they had multiple short-term absences due to sick leave. A related problem applies to employees with a §56-agreement, i.e. their employers are entitled to reimbursement of sickness benefits from day one; such employees may be misclassified as u-WPS in the current study due to sick leave registrations exceeding actual duration. These possible misclassifications are considered non-differentiated since they are not suspected to be associated with the exposure (i.e. the prognostic variables). Unfortunately, the data do not offer any insight as to the possible distribution of §56-agreements in the current study. However, explorative *post-hoc* analyses were performed in which single weeks of sickness benefit reimbursement were considered as §56-agreements, i.e. regarded as working weeks. This resulted in the distribution of s-WPS/u-WPS changing from 100/68 to 102/66 (data not shown). It did not change the results of the study.

Sixth, the limited sample size implies a risk of type II errors. That is, important prognostic factors may not be discovered as statistically significant owing to the limited sample size. Other factors that could affect the prognosis for work outcomes include fear avoidance beliefs [2, 3, 49] and physical workload [1, 2, 39]. These factors were not isolated for analysis in the current study since they were covered by the ÖMPSQ score and we wished for all variables to be as mutually exclusive as possible. Furthermore, the aim was not to investigate a complete list of all possible factors affecting WPS prognosis but rather to explore the contributions of demographic, patient-reported, clinical and MRI variables for which purpose we believe the current sample size was adequate.

## Conclusions

In this study of sickness absentees with neck or shoulder pain, clinical and MRI information provided no additional information for the prediction of work participation compared with only demographic and patient-reported information.

Though Model 2 performs similarly to Models 3 and 4, there are limitations with respect to discriminating between those with the lowest and highest chance of s-WPS. This means that clinical applicability requires caution if the aim is identification of those who have the lowest/highest chances of s-WPS. The results do, however, provide valuable knowledge to clinicians both in the assessment of work prognosis and in dialogue with patients and other stakeholders: prediction should primarily be based on demographic and patient-reported information, not on clinical and MRI findings.

Due to the exploratory nature of the current study, the generalizability of findings needs to be assessed in independent studies, preferably in both primary and secondary care settings.

## Data Availability

The datasets used and/or analysed during the current study are available from the corresponding author on reasonable request.

## Abbreviations

AUC: Area Under the Curve
DREAM: Danish Register for Evaluation of Marginalization
MRI: Magnetic Resonance Imaging
NPV: Negative Predictive Value
ÖMPSQ: Örebro Musculoskeletal Pain Screening Questionnaire
PPV: Positive Predictive Value
RCT: Randomized Controlled Trial
RTW: Return To Work
s-WPS: Successful Work Participation Score
u-WPS: Unsuccessful Work Participation Score
WPS: Work Participation Score
